# Systematic Reverse Engineering of Network Topologies: A Case Study of Resettable Bistable Cellular Responses

**DOI:** 10.1371/journal.pone.0105833

**Published:** 2014-08-29

**Authors:** Debasish Mondal, Edward Dougherty, Abhishek Mukhopadhyay, Adria Carbo, Guang Yao, Jianhua Xing

**Affiliations:** 1 Department of Biological Sciences, Virginia Bioinformatics Institute, Virginia Polytechnic Institute and State University, Blacksburg, Virginia, United States of America; 2 Department of Genetics, Bioinformatics and Computational Biology Ph. D program, Virginia Bioinformatics Institute, Virginia Polytechnic Institute and State University, Blacksburg, Virginia, United States of America; 3 Department of Physics, Virginia Bioinformatics Institute, Virginia Polytechnic Institute and State University, Blacksburg, Virginia, United States of America; 4 Department of Computer Science, Virginia Bioinformatics Institute, Virginia Polytechnic Institute and State University, Blacksburg, Virginia, United States of America; 5 Nutritional Immunology and Molecular Medicine Laboratory and Center for Modeling Immunity to Enteric Pathogens, Virginia Bioinformatics Institute, Virginia Polytechnic Institute and State University, Blacksburg, Virginia, United States of America; 6 Department of Molecular & Cellular Biology, University of Arizona, Tucson, Arizona, United States of America; 7 Beijing Computational Science Research Center, Beijing, China; Fondazione Edmund Mach, Research and Innovation Centre, Italy

## Abstract

A focused theme in systems biology is to uncover design principles of biological networks, that is, how specific network structures yield specific systems properties. For this purpose, we have previously developed a reverse engineering procedure to identify network topologies with high likelihood in generating desired systems properties. Our method searches the *continuous* parameter space of an assembly of network topologies, without enumerating individual network topologies separately as traditionally done in other reverse engineering procedures. Here we tested this CPSS (continuous parameter space search) method on a previously studied problem: the resettable bistability of an Rb-E2F gene network in regulating the quiescence-to-proliferation transition of mammalian cells. From a simplified Rb-E2F gene network, we identified network topologies responsible for generating resettable bistability. The CPSS-identified topologies are consistent with those reported in the previous study based on individual topology search (ITS), demonstrating the effectiveness of the CPSS approach. Since the CPSS and ITS searches are based on different mathematical formulations and different algorithms, the consistency of the results also helps cross-validate both approaches. A unique advantage of the CPSS approach lies in its applicability to biological networks with large numbers of nodes. To aid the application of the CPSS approach to the study of other biological systems, we have developed a computer package that is available in Information S1.

## Introduction

Systems biology studies how a biological system evolves to perform certain function(s), *i.e*., the design principles of the system [1]–[3]. Reverse engineering is a computational approach to deduce biological network structures responsible for given properties [4]–[15]; it addresses situations that while certain dynamic properties of a biological system have been observed, the underlying mechanisms are unknown or incomplete. To reverse engineer, one can perform a thorough *in silico* search to enumerate all possible network topologies leading to the dynamic feature at question, then identify the most plausible network topologies. To help illustrate the basic strategy, let's consider a simple three-node network. There are 9 possible links in the full graph including self-regulatory links. Each link has three possibilities: activation, inhibition, or no presence. Consequently, there are a total of 3^9^ possible network topologies. As a common reverse engineering procedure, one can model each network topology against a collection of random parameter sets, and evaluate the robustness of each network topology (i.e., the proportion of the parameter sets allowing each topology to produce the desired dynamic feature). This procedure, which we name as ITS (individual topology search), has been successfully adopted to analyze several important biological processes including segment polarity [7], perfect adaption [8], and bistability [13]. However, the ITS approach is difficult to apply to systems with large numbers of nodes for two reasons. First, the number of network topologies grows dramatically with the number of nodes. For example, the total numbers of network topologies for 4-node and 5-node systems are 3^16^ = 4.3×10^7^ and 3^25^ = 8.5×10^11^, respectively (compared to 3^9^ = 2.0×10^4^ for a 3-node system). Second, since the number of model parameters increases with the number of nodes, the fraction of the parameter space leading to the desired feature (the ‘*good*’ region) decreases exponentially. For example, suppose that the parameter-space dimension is *N*, and that for each parameter, half of the parameter range falls into the good region, the maximum fraction of the good region with the parameter space is *1/2^N^*.

Recently, we developed a computational approach to overcome the above difficulties [4]. Instead of modeling individual network topologies separately, our approach searches the continuous parameter space that defines an assembly of individual topologies derived from the full network (with the presence/absence and the strength of each network link indicated by corresponding parameter values). Applying this new CPSS (continuous parameter space search) approach, we notice that the good regions are typically sparse and isolated within the parameter space [4]. To efficiently recover these good regions, we employed a two-stage sampling procedure. In the first stage, techniques such as importance sample [16] or genetic algorithm [17], [18] are used to locate isolated good regions with a small number of good parameter sets (“seeds”) for each region. In the second stage, the seeds are used to perform random walk (importance sampling) constrained within each isolated good region. This two-stage procedure is effective to recover the good regions, even when they account for a tiny fraction (e.g., as low as 10^−7^) of the whole parameter space. The CPSS method can therefore effectively search exponentially growing topological and parameter spaces accompanied with larger networks. For example, we have recently applied this CPSS approach to study a network with 10 nodes and 64 parameters [19].

How well do the results obtained from the two different approaches, CPSS and ITS, agree with each other? The present work aims to address this question. Here we first present a detailed and improved CPSS procedure, we then test the CPSS procedure on a previously analyzed problem based on ITS – the resettable bistability of an Rb-E2F gene network that controls the mammalian cell cycle entry [13]. We find that the CPSS and ITS approaches, based on different mathematical formulations and search algorithms, generate consistent search results. This consistency demonstrates the overall effectiveness of reverse engineering approaches (CPSS and ITS) in uncovering network design principles. To aid the application of the CPSS approach, we also develop a computer package that can be conveniently used to reverse engineer other biological networks, especially those with large numbers of nodes with which the ITS approach becomes inefficient.

## Methods

### (*a*) Problem setup and outline of the CPSS procedure

**Figure 1:**
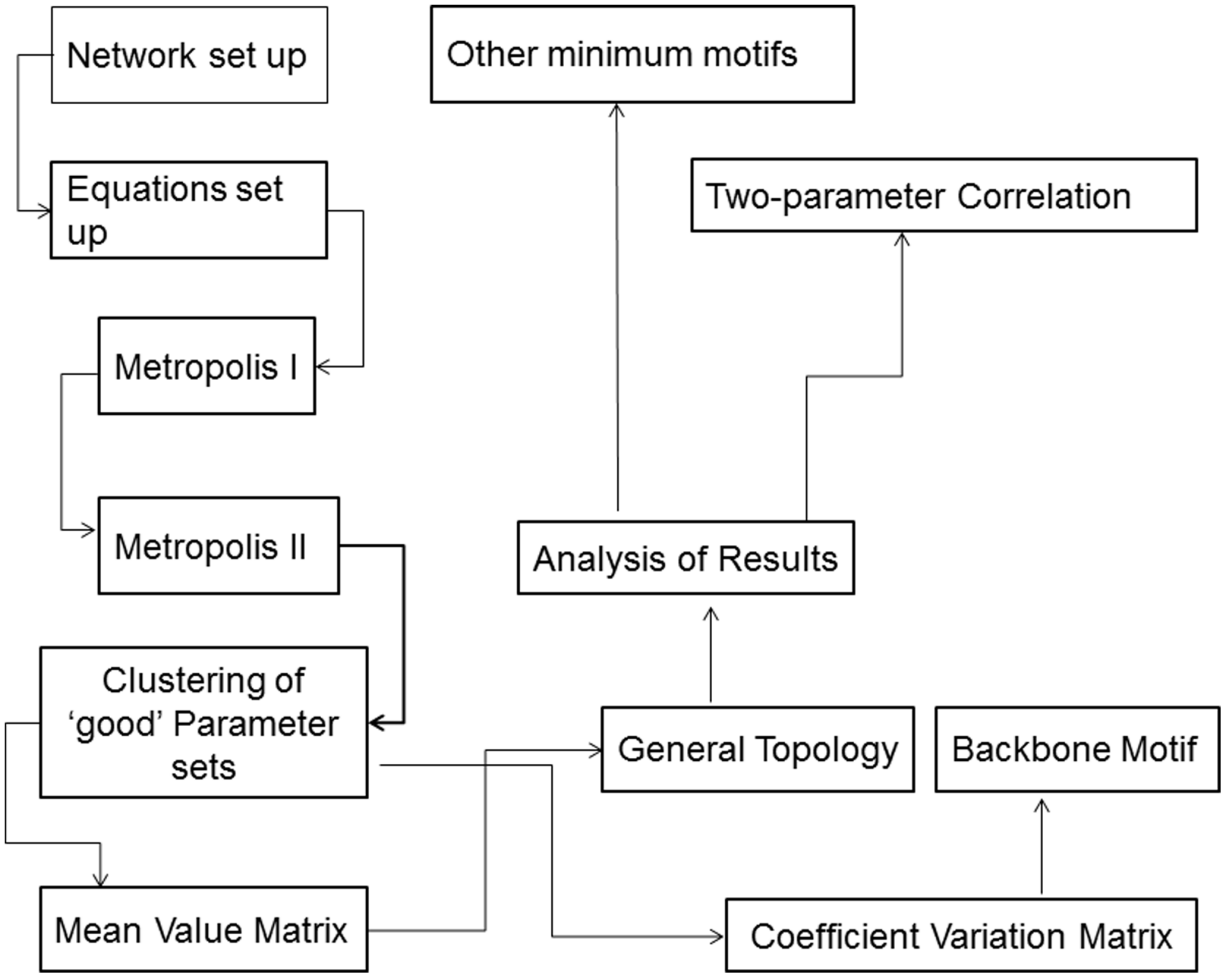
Flowchart describing the CPSS method to determine and analyze network topologies underlying a given dynamic property.

The steps below describe our CPSS procedure (with Figure 1 showing the flow chart).

- Set up a network (with a selected number of nodes) associated with the concerned dynamical property, based on experimental evidence and literature.
- Construct mathematical equations to describe the interactions among nodes in the network. We use the Wilson-Cowan function [4], [19]–[21] for its mathematical flexibility.
- Define the search criteria for the dynamic property under consideration (*e.g.*, bistability).
- Determine the dimension of the parameter space (*N*) and perform a two-stage search to get ‘good’ parameter sets that fulfill the criteria in (iii).
- Determine the optimal number of clusters formed by the good parameter sets.
- For each cluster, construct the mean value (MV) matrix and the coefficient variation (CV) matrix (see detailed discussions later) to determine “mean” network topology and backbone motifs responsible for the desired dynamic property.
- Analyze the roles of individual network links in generating the desired dynamic property.

**Figure 2:**
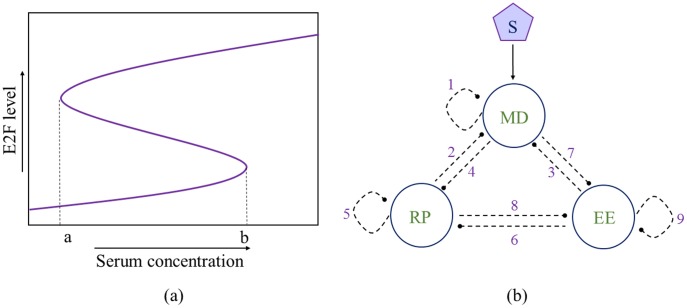
Schematic representation of Rb-E2F network. (a) Resettable bistability of the Rb-E2F network. Points on the x-axis (*a* and *b*) define the left and right boundaries of the bistable region. (b) A coarse-grained 3-node Rb-E2F network following the setup of Yao *et. al*. [13], [22]. S: serum input; MD: linear signaling cascade consisting of Ras, Myc and CycD/cdk4,6; RP: Rb family proteins; EE: E2F activators and CycE/cdk2.

In this work, the dynamic property we study is the resettable bistability of an Rb-E2F gene network that controls the mammalian cell cycle entry. Yao *et al.* demonstrated that the Rb-E2F gene network functions as a bistable switch (Figure 2a) [13], [22]. The Rb-E2F bistable switch converts graded and transient serum growth signals into an all-or-none E2F activation, which controls the quiescence-to-proliferation transition of mammalian cells. This Rb-E2F bistable switch is resettable: that is, the activated switch can be fully shut off when the strength of serum signals is reduced below a threshold (point *a* in Figure 2a) [13], [22]. The Rb-E2F gene network also exhibits other dynamic properties such as the biphasic response of E2F to MYC stimulation [23], and likely coordinates the implementation of different dynamic properties by constraining associated network parameters [24].

To identify the network topology responsible for the emergent resettable bistability in the Rb-E2F gene network, Yao *et al.* [13] took a reverse-engineering approach. They coarse-grained the Rb-E2F network into a 3-node circuit and examined what network topologies among possible link combinations lead to robust resettable bistability. Here we follow their setup of the coarse-grained network, combining all E2F activators and CycE/cdk2 into one node EE, all Rb family proteins into another node RP, with the third node MD representing the linear signaling cascade consisting of Ras, Myc and CycD/cdk4, 6. In this 3-node network, 9 possible links exist (Figure 2b). We investigate two cases of the network. First (Case I), to be consistent with the study of Yao *et. al.* [13], which is based on known network links in the literature, we do not consider the link from RP to MD (link 2) and self-regulatory links of MD and RP (links 1 and 5, respectively; Figure 2b). Second (Case II), we examine the full graph that contains all the 9 possible links (see Text S1).

### (*b*) Mathematical description

We use the following mathematical formalism of ordinary differential equations (ODEs) to describe the 3-node Rb-E2F network:
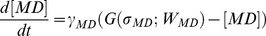
(1a)

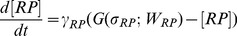
(1b)

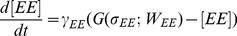
(1c)


Where 
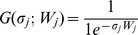
 and 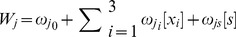
(1d)


Here, *G(σ_j_; W_j_)* is a generic ‘*sigmoidal*’ function with steepness (slope at *W*_j_ = 0) that increases with σ_j_ for the *j* ^th^ species. For present study, both *i* and *j* can be any one of the three nodes, MD, RP, or EE. We set a range for σ_j_ between 1–10. *W*_j_ denotes the overall influence of the network on node *j*. The coefficient ω_ji_ measures the strength and direction of the influence of the *i* ^th^ species on the *j* ^th^. The term ω_jS_ specifies whether a species *j* is affected by the input signal (serum concentration), and assumes a value of 1 for node MD and 0 otherwise. Each ω_ji_ is a real number between [−1, 1], with a positive value for activation and negative value for inhibition. Thus, the sign pattern (−, 0, +) of the ‘weight’ matrix ω_ji_ defines the topology of the network. The term ω_j0_ determines whether the *j* ^th^ node is ‘on’ or ‘off’ when all other input signals are at 0. The parameter γ_j_ determines how quickly each species approaches its steady state value. The value of γ_j_ does not affect the steady-state behavior of the network but controls the non-stationary network dynamics. We denote the concentration of a species *x* by [x]. We construct the ODEs for the 3-node network in a formulation similar to previous models [4], [20], [25], [26]. We refer [10], [27]–[29] for more detailed discussions and applications of the formalism.

For case I, the model described by Eq. 1a–d contains 15 parameters: 6 *ω_ji_*, 3 *γ_j_*, 3 *σ_j_* and 3 *ω_j0_*. In the following studies we fix 7 parameters constant: we set *γ_EE_* = *γ_MD_* = *γ_RP_* = 1.0 since they have no effect on the steady state behavior; we set *ω_MD0_* = *ω_EE0_* = −0.5 and *ω_RP0_* = 0.5 so that [EE]_ss_∼0 in absence of input signals (to be consistent with the experimental observation); we also set *σ_EE_* = 5.0 as a moderate value for the sigmoidicity of the output response of node EE. Therefore, our search is in an 8-dimensional parameter space. For Case II, we consider three additional parameters (*ω_MDRP_*, *ω_MDMD_*, and *ω_RPRP_*) and perform the search in an 11-dimensional parameter space.

### (*c*) Determination of condition for resettable bistability

For each given parameter set, we calculate the steady state concentrations of EE node ([EE]_ss_) under a set of input serum concentrations uniformly distributed between 0 and 10. At each input signal level, we use two different sets of initial conditions, corresponding to the quiescence state (EE^OFF^: [EE] = [MD] = 0.001, [RP] = 1.0 and the proliferation state (EE^ON^: [EE] = [MD] = 1.0, [RP] = 0.001). Temporal evolution of each node is simulated using the deterministic improved Euler method [30], with sufficiently long simulation time to ensure that the system reaches steady state. To determine whether a network topology creates resettable bistability with a given parameter set, we follow the same three criteria used in Yao *et al.* (that yielded top candidate topologies consistent with further experimental tests) [13]:

- Being a switch: [EE]_ss-max_ -[EE]_ss-min_>λ. Here, [EE]_ss-max_ and [EE]_ss-min_ denote the maximum and minimum values of the steady-state EE concentration, respectively, simulated with the EE^OFF^ initial condition for the entire serum input range; the threshold λ is set to 0.1.
- Being bistable: Δ[EE]_ss_ = [EE]_ss_^ON^-[EE]_ss_^OFF^>0.1×([EE]_ss-max_ - [EE]_ss-min_) for at least two of the input levels tested (except for the saturation level [S]_max_ = 10, where the system should be monostable). Here, [EE]_ss_^ON^ and [EE]_ss_^OFF^ denote the steady-state EE concentrations obtained with the EE^ON^ and EE^OFF^ initial conditions under a given input level, respectively.
- Being resettable: Δ [EE]_ss_→0, when [S]→0.

If all three criteria are met, we refer the associated parameter set as a ‘good’ parameter set. We define a binary score function for the k^th^ parameter set: W_k_ = −1 if all the criteria i–iii for resettable bistability are fulfilled; W_k_ = 0 otherwise.

In addition, to address the concern that the identified candidate topologies may be sensitive to the chosen threshold values in the above criteria i–iii, we also perform searches with another constraint following Yao *et al.* [13] and others [31]. This extra independent constraint, R^0^, is defined as R^0^ = [S_off_]^max^/[S_on_]^min^, with [S_off_]^max^ and [S_on_]^min^ correspond to the right and left boundaries of the bistable region (*b* and *a*, Figure 2a), respectively. Thus, R^0^ is positively correlated with the width of the bistable region. The comparison of the identified candidate topologies with or without this extra constraint R^0^ helps evaluate the “robustness” of our model predictions. We consider a network topology with R^0^≥3.0 as a topology that fulfills the criterion iv; correspondingly, we use a score function to define good parameter sets, W^c^ = 3.0×W_k_. For convenience of discussion, we refer the searches with the criteria i–iii as “*Normal*”, and those with criteria i–iv as “*Constrained*”. Also, all following discussions refer to Case I (see subsection *a* and *b* of *Methods* section for definition) unless otherwise noted.

### (*d*) Metropolis sampling algorithm

Our goal is to sample an 8-dimensional parameter space that is bounded and continuous. The sampling algorithm needs to search the parameter space thoroughly and generate sample parameter sets that are statistically unbiased and significant. Our strategy is a random walk method based on the Metropolis Algorithm [32] using the following scheme:

- Choose an arbitrary initial 8-dimensional parameter set θ_0_ and determine its score: W_0_ = −1.0 for ‘good’ set and W_0_ = 0.0 otherwise.
- Generate parameter set θ_k+1_ from θ_k_ by θ_k+1_ = θ_k_+λζ; λ ( = 0.025 in this work) specifies the maximum displacement per step, and ζ is a vector of random numbers with uniform distribution between −1.0 and 1.0.
- Compute W_k+1_. If W_k_≥W_k+1_, accept the step from k to k+1. If W_k_<W_k+1_, accept the step from k to k+1 with probabilityρ.
- Update θ_k_. If k is larger than a maximum step number (N_max_), stop. Otherwise, return to step (ii).

We implement this strategy in two stages. In Stage I, we set ρ = 0.01, so that the random walk has a tendency to stay in ‘good’ regions of parameter space, but it can also jump out of a good region and searches randomly until it falls into another good region (or back into the previous region). A random walk with up to 10^7^ (N_max_) steps is performed in Stage I until a good parameter set is identified (W_k_ = −1 for the ‘*Normal*’ parameter search, and W^c^ = −3.0 for ‘*constrained*’ parameter search). Under either situation (*Normal* or *Constrained*), we repeat the Stage I from different starting points until 30–50 ‘*good*’ parameter sets are found.

In Stage II, each ‘*good*’ parameter set obtained from Stage I serves as a “seed” for performing random walk within the ‘*good*’ parameter region (with ρ = 0 to constrain the random walk to the same good region). The purpose of Stage II is to generate a large sample of good parameter sets that occupy each of the different good regions within the parameter space. We set N_max_ = 10^7^ for each run and the random walks are sampled every 100 steps, generating ∼10^3^–10^5^ ‘*good*’ parameter sets from each seed.

We note that the introduction of the two-stage Metropolis sampling algorithm described above strengthens the efficiency of the CPSS method. Stage I sampling helps find seeds for ‘good parameter regions’ following random walks. After the detection of good seeds in Stage I, Stage II sampling only explores the volume element of a ‘good region’ of each seed in the N-dimensional parameter space. This two-stage method directs the search to the “functional” (or “good”) regions, without spending much time on regions not giving rise to the desired properties (resettable bistability in this case). Therefore, the computational efficiency of the CPSS method would be significantly improved over the ITS method, especially in high-dimensional systems.

### (*e*) Clustering of the good parameter sets

The good parameter sets obtained from the two-stage search may form either a single cluster or several different clusters. The parameter sets within each cluster correspond to network topologies sharing certain common features. Similar to our previously developed CPSS method [4], we apply the K-means clustering algorithm [33], [34] (K = 2 to 12) to identify possible clusters of good parameter sets. In addition, here we perform an extra procedure to determine the optimal number of K. That is, we calculate the mean silhouette coefficient values [35], [36] for each K; the largest mean silhouette coefficient value suggests the optimal K number. If it happens that the optimal K equals 2, we would manually check whether the parameter sets are indeed distributed in two distinct clusters or they correspond to a single cluster (see Text S1 for details).

### (*f*) Discretization of continuous parameter matrix into topology matrix

In order to identify the topological feature underlying the resettable bistable mechanism, we coarse grain the continuous interaction coefficient *ω_ji_* (Eq. 1d) into a discretized topological matrix τ_ji_. In the topological space, τ_ji_ is only described by (−, 0, +), representing inhibition, no interaction, and activation, respectively. A cut-off value (0.1) is used to perform the discretization, following the rules below:

- If −0.1≤ω_ji_≤0.1, τ_ji_ is considered as 0 (no interaction).
- If ω_ji_>0.1, τ_ji_ is considered as ‘+’ (activation).
- If ω_ji_<−0.1, τ_ji_ is considered as ‘−’ (inhibition).

### (*g*) Statistical method to identify the mean motif and a backbone motif

For each cluster identified in step e, we perform the following procedure to get a mean motif:

- Calculate the mean of each interaction coefficient ω_ji_ among all good parameter sets.
- Map the mean value (MV) matrix into a topological matrix τ_ji_ using parameter discretization (step *f*).

We also define a backbone motif as the motif with the fewest number of non-zero

*ω_ji_* that is shared by the maximum number of resettable bistable network topologies within a single cluster, and that by itself is sufficient to generate resettable bistability. Identification of backbone motifs helps to define the core mechanism of resettable bistability. To identify a backbone motif, we first calculate the CV matrix for each cluster. As the CV matrix measures the dispersion of the data along the range of each parameter *ω_ji_*, a large CV value of a given *ω_ji_* suggests that the dispersion around the mean interaction strength of the corresponding link is large and thus the said link is not essential to be part of a backbone motif. Only links with CV < Cut-off should be part of a backbone motif. For CV > Cut-off, we set τ_ji_ = 0 in the backbone motif. To determine an optimal value of Cut-off we follow a strategy described as follows. As Cut-off decreases, the corresponding motif becomes simpler and therefore more network topologies contain this motif; yet, this motif cannot be too simple to lose resettable bistability. Therefore, there exists an optimal Cut-off value so that the corresponding motif has the minimal topology that is sufficient to generate resettable bistability, and that the fraction of network topologies containing this backbone motif within a cluster is the highest.

### (*h*) Software implementation

**Figure 3:**
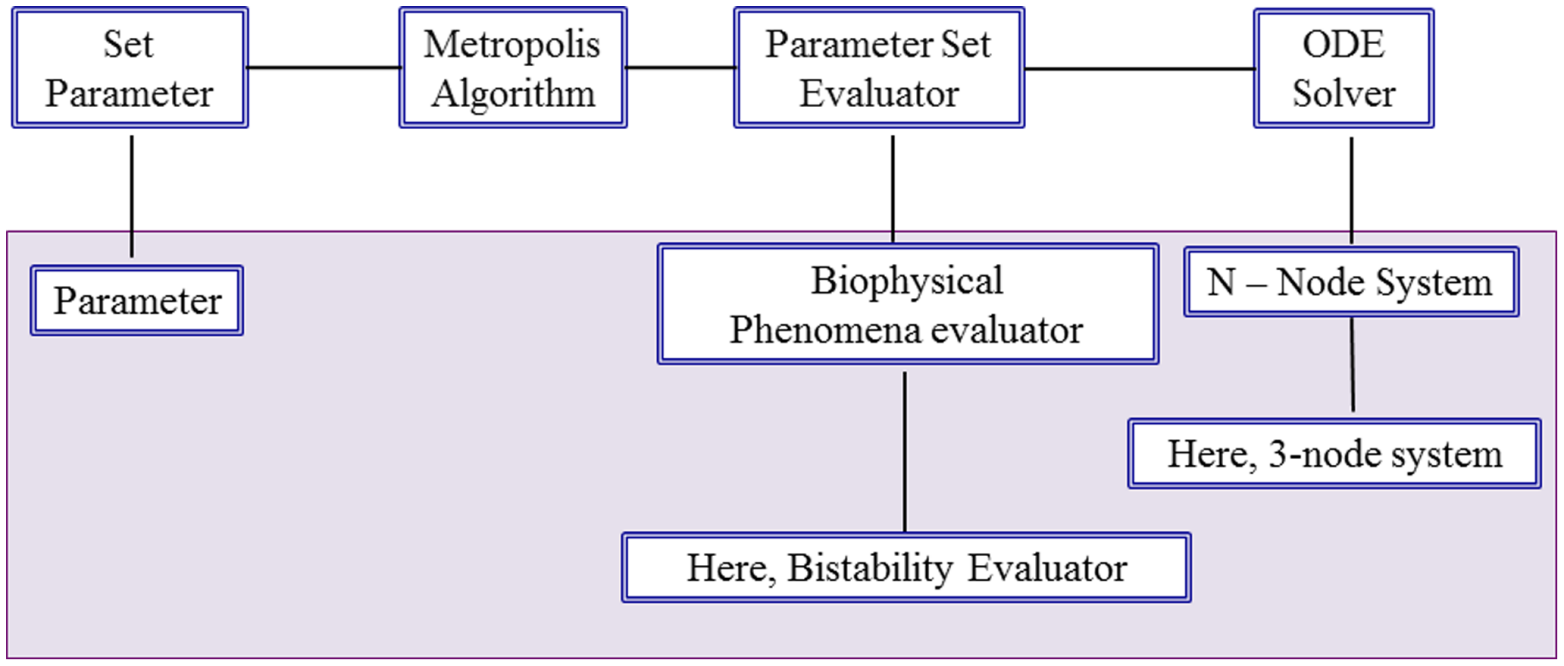
CPSS software implementation. The major functional modules (above the gray box) are application-independent objects and will not need to be changed for a new application. The modules within the gray box are application-specific objects; they inherit all properties of their “parent” objects, which help minimize the amount of new coding needed. For example, the present study uses a bistability evaluator (under “network feature evaluator”) and a 3-node system (under “ODE solver”). A different feature evaluator (e.g. adaptation) and a network with larger number of nodes can be similarly implemented for a new study.

To help automate our previously developed CPSS procedure and improve its computational efficiency, as well as to aid the application of CPSS to study a broad range of biological networks, we construct an integrated software package with the following features. (1) High flexibility: by implementing the CPSS procedure in several independent and interchangeable functional modules (Figure 3), the need for recoding is minimized to perform CPSS in applications involving different network topologies, dynamic properties, and different mathematical formulations. (2) User friendly: the software is implemented with minimal required human-in-the-loop interaction. (3) Computational efficiency: we use the C++ programming language to implement the search algorithm to minimize execution time, which can perform billions of CPSS iteration steps within few days to weeks depending on the dimensionality of the problem. An assembled package consisting the entire CPSS program is included in Information S1.

## Results

At stage I, we first search the Rb-E2F ODE system (Eq 1a–d) with 35 arbitrary initial parameter sets, and identify 35 ‘good’ parameter sets. For each of these 35 ‘seeds’, we then perform the stage II search with 10^7^ Metropolis steps. We obtain a total of ∼2.0×10^5^ good parameter sets. We perform the K-means clustering (see subsection *e in Methods* section) and find that all good parameter sets form a single cluster in the parameter space (See section T1 in the Text S1).

### (a) Structure of the mean motifs and backbone motifs

**Figure 4:**
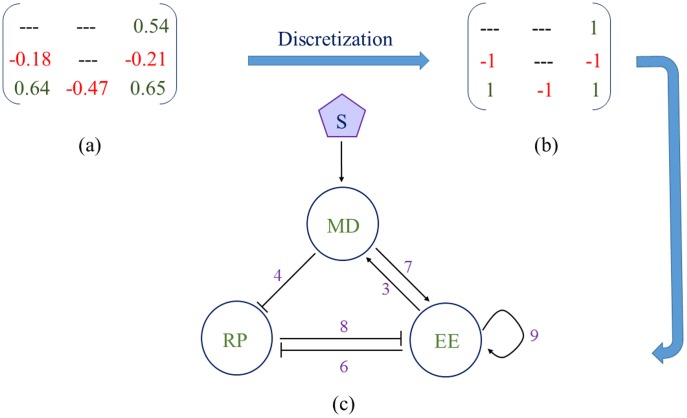
Mean network topology underlying the resettable bistability. (a) Mean value matrix consisting all six network links. (b) Topology matrix after discretization of Mean value matrix. (c) Mean network topology obtained from the topology matrix.

Figure 4a gives the mean value (MV) weight matrix denoting different link strengths. From the MV matrix we construct discrete topology matrix (Figure 4b) following the discretization principle (subsection *f* in *Methods* section). Figure 4c depicts the mean network topology underlying the resettable bistability, which contains three positive feedback loops: a positive feedback between MD and EE (links 3–7), a double negative feedback between RP and EE (links 6–8), and an EE Self-activation (link 9). This mean network topology (Figure 4c) is consistent with the most robust topology in generating a Rb-E2F bistable switch as identified in the study of Yao *et al.* [13].

**Figure 5:**
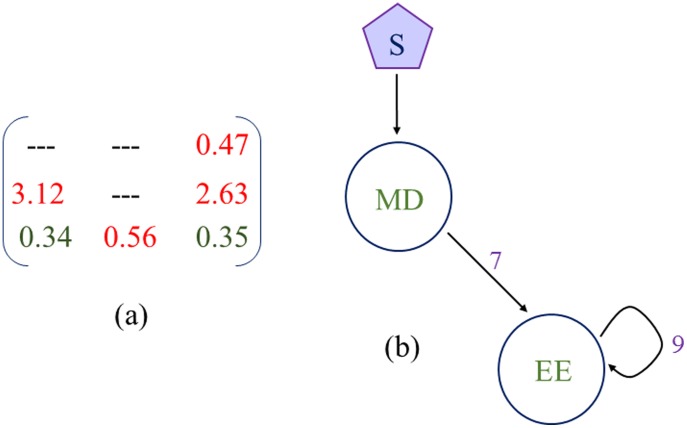
Backbone motif underlying resettable bistability. (a) Coefficient Variation matrix. (b) The backbone motif obtained from the Coefficient Variation matrix.

To determine backbone motifs underlying the resettable bistability, we construct the CV matrix from the good parameter sets (Figure 5a) and set a CV cut-off value 0.36 corresponding to a high (more than 0.95) sample ratio. A sample ratio of a given link is a fraction of *good* parameter sets which contain the said link in network topologies within a cluster. The identified backbone motif (Figure 5b) contains links 7 and 9. That is, this 7–9 motif is a minimum circuit that is shared by most resettable bistable network topologies; this motif is also sufficient to generate resettable bistability by itself. This backbone motif is one of the minimum motifs discovered by Yao *et al.* using a different approach, ITS [13]. In addition, we identify several robust minimum motifs, which are defined as topologies with *high* occurrence probability in the entire good parameter sets. We determine the fraction of good parameter sets corresponding to individual minimum motifs within the cluster. Table 1 gives a few top minimum motifs with 2, 3, or 4 links. These top minimal motifs are also present amongst the top minimum topology list in the study of Yao *et al.* [13], which signifies the consistency of present results.

**Table 1:** Robust minimum motifs underlying resettable bistability for Normal situation.

Nature of the Link	Occurrence Probability	Present as Minimal model in [13]?
7----- 9	98.9	Yes (as 7----9a)
3 ----- 7	98.1	Yes (as 2----7)
3---4---8	66.0	Yes (as 2---5---6)
4---8---9	66.6	Yes (as 3---6---9)
4---6---8	55.4	Yes (as 3---5---6)
6---7---8	68.6	Yes (as 5---6---7)
4---6---8---7	55.4	Yes (as 3---5---6—7)
4---9---8---7	66.6	Yes (as 3---6 --- 7---9)
4---8---3---7	65.9	Yes (as 2---3---6---7)

### (b) Functional role of the link 3

**Figure 6:**
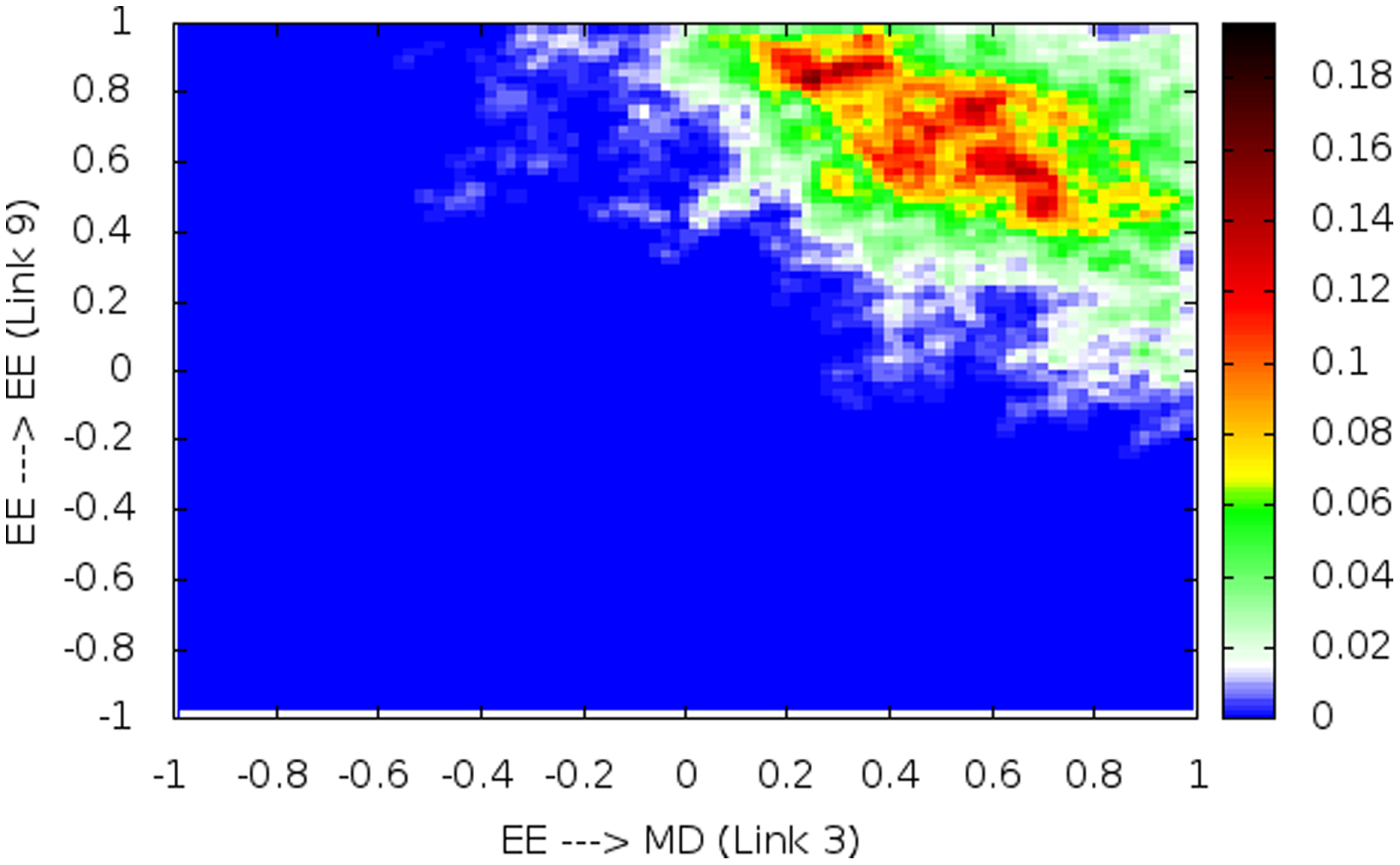
Pairwise correlation between links 3 and 9: 2D correlation heat map. The x axis denotes the link from EE to MD (link 3); the y axis denotes the link from EE to itself (link 9). The value on each axis denotes the link strength, with the positive and negative segments indicating activation and repression links, respectively. Color bar on the right: the fraction of ‘good’ parameter sets (supporting resettable bistability).

**Figure 7:**
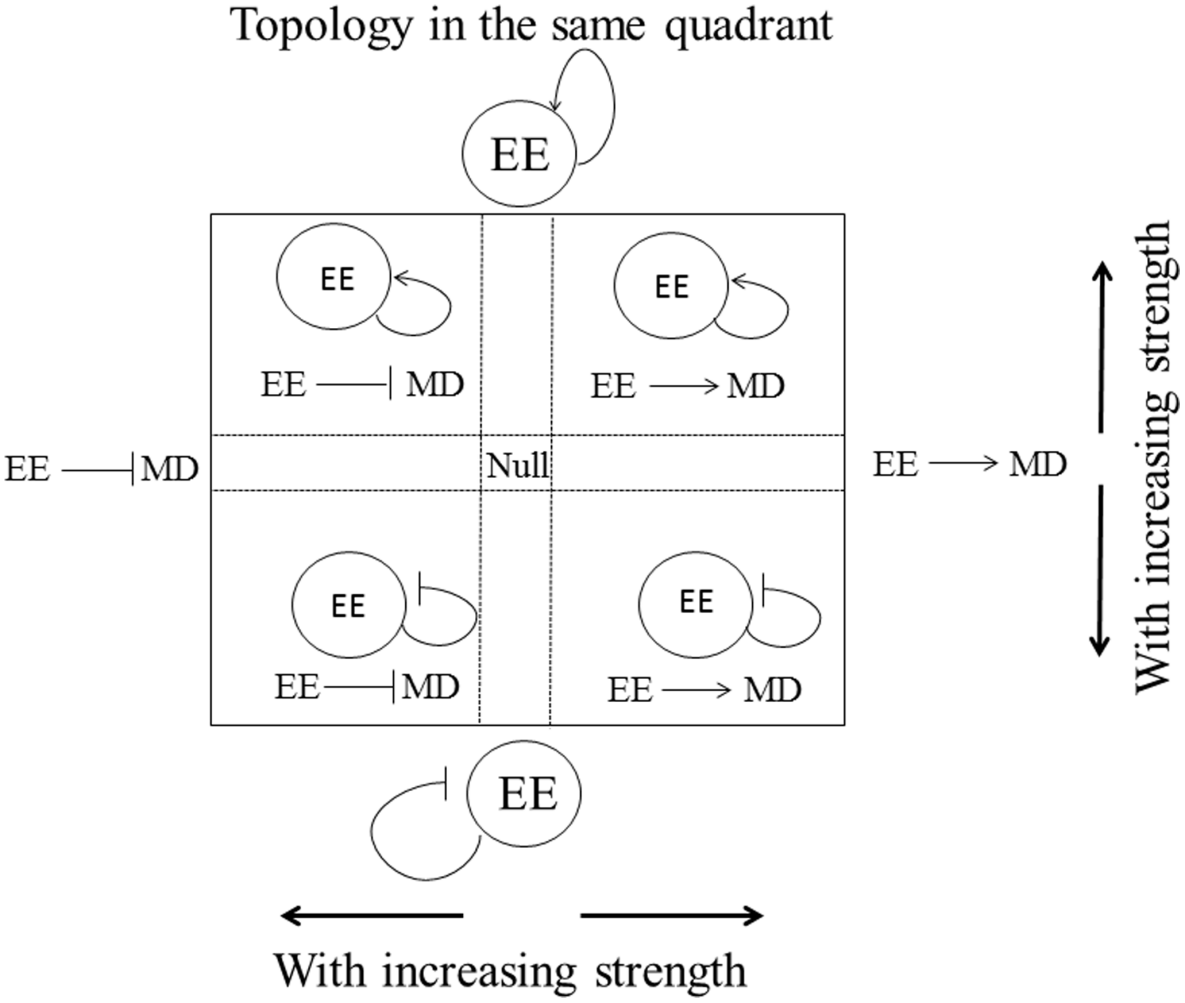
Pairwise correlation between links 3 and 9: Diagrams of link combinations that correspond to the heat map in **Figure 6**.

Positive feedback regulation is required for generating bistability [1]. The mean network topology underlying resettable bistability identified in this work (Figure 4c) contains three positive feedback loops. Link 3, which is part of the double-positive feedback loop of links 3-7, is present in the most robust topology for bistability but not in the most robust topology for resettable bistability in Yao et. al. [13]. To analyze this discrepancy, we examine the correlation between link 3 and other links in the mean topology. We found within certain parameter ranges, the presence of link 3 (and thus the positive feedback loop 3-7) facilitates resettable bistability. For example, the heat map in Figure 6 shows that the “good” data points are in the upper right quadrant, indicating both links 3 and 9 should be activation links to support resettable bistability (Figure 7). Furthermore, there exists a negative correlation between the strengths of links 3 and 9 among the good data points; meanwhile, an intermediate strength link 3 (>0.2, <0.6) is favorable. These results suggest that the strength of link 3, when properly constrained, helps maximize the creation of bistability while maintaining its resettability.

### (c) “Lumped parameter” effects

We note that the above mentioned negative correlation between links 3 and 9 to create good data points (Figure 6) provides an example of lumped parameter effects. That is, sometimes a combinatorial effect of two or more parameters, instead of individual parameter values, dictates network dynamics. The lumped parameter effects can be explained by the nature of the modeling equations (Eq. 1(a-d)). Eq. 1d explains that the activation of the j^th^ species is dependent on the overall net input W_j_. As W_j_ combines inputs from all three regulating nodes, any change in one parameter, say ω_jMD_, can be compensated by a change in the other two parameters (ω_jEE_ or ω_jRB_, or both). Such parameter compensation expands the region of parameter space that supports resettable bistability, and thus enhances the robustness of the model.

### (d) Consistent results from the “Constrained” and “Normal” situations

**Figure 8:**
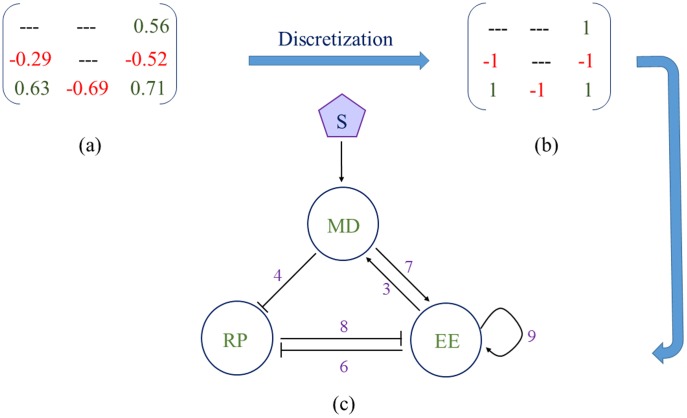
Mean network motif for resettable bistability under the Constrained situation. (a) Mean value matrix consisting all six network links. (b) Topology Matrix after discretization of the Mean value matrix. (c) Mean network topology, which is responsible for resettable bistability under the Constrained situation.

**Figure 9:**
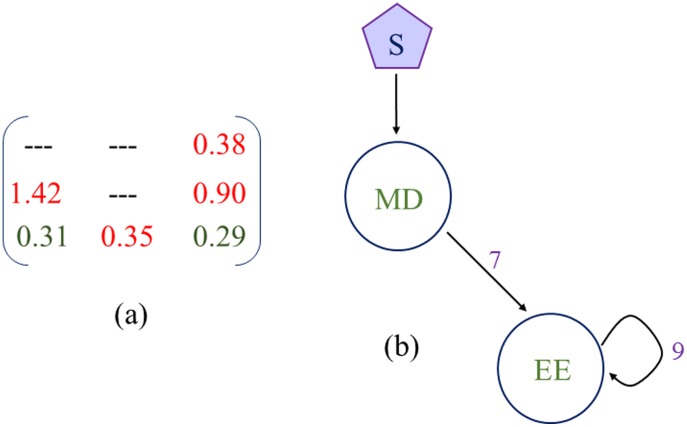
Backbone motif underlying resettable bistability under the Constrained situation. (a) Coefficient Variation matrix. (b) Backbone motifs obtained from the Coefficient Variation matrix. See text for details.

We also perform the CPSS search with the bistable region constraint R^0^ (see subsection c in Methods section) on the resettable bistability. The mean network topology identified (Figure 8) shows that the additional constraint (R^0^≥3) does not change the mean motif structure obtained in the ‘*Normal*’ situation. This is also consistent with the observation in [13]. A comparison of the MV matrix between the ‘*Normal*’ and ‘*Constrained*’ situations suggests that most link strengths in the mean network topology under the Constrained situation are increased (compared to those under the Normal situation). Meanwhile, the backbone motif and the robust minimum motifs in the Constrained situation are consistent with those found in the Normal situation (Figure 9; Table 2). This consistency suggests that the CPSS-identified candidate topologies are not sensitive to the chosen threshold values in the search criteria (i-iii, subsection c in Methods section).

**Table 2:** Robust minimum motifs underlying resettable bistability for Constrained situation.

Nature of the Link	Occurrence Probability	Present as Minimal model in [13]?
7----- 9	99.6	Yes (as 7----9a)
3 ----- 7	99.0	Yes (as 2----7)
3---4---8	87.3	Yes (as 2---5---6)
4---8---9	87.4	Yes (as 3---6---9)
4---6---8	85.5	Yes (as 3---5---6)
6---7---8	89.2	Yes (as 5---6---7)
4---6---8---7	85.5	Yes (as 3---5---6—7)
4---9---8---7	87.4	Yes (as 3---6 --- 7---9)
4---8---3---7	87.3	Yes (as 2---3---6---7)

### (e) CPSS with all possible links in the Rb-E2F network (Case II)

In Text S1, we also present analysis of Case II, which allows the presence of link 1, 2 and 5 (Figure 2b). Notably, results from our CPSS analysis *posteriori* reveal that links 1 and 5 (self-interaction links of MD and RP nodes, respectively) are not favorable, indicating a functional selection pressure against evolving these two links in the Rb-E2F network. On the other hand, the additional link 2 (inhibition link from RP to MD) is included in the identified mean motif, while the backbone motif obtained from CV matrix analysis is unaltered (see section T2 in Text S1). Thus, an inhibition from RP to MD (forming a double negative feedback loop with link 4, Figure 2b), if evolved in the Rb-E2F network, can facilitate the resettable bistability. The link 2, however, is not essential for the generation of resettable bistability, as it is not present in the backbone motif obtained from the CV matrix analysis.

## Discussion

In this work we present a thorough comparison of two different reverse engineering approaches, CPSS and ITS. The ITS approach used by Yao *et al.* [13] examines each possible network topologies individually, and adopts the Hill-type function form. The present one, CPSS, explores the continuous parameter space, and adopts the Wilson-Cowan type function form. Our analysis shows that the two approaches give consistent results on the mean network topology and the backbone motifs underlying resettable bistability in the Rb-E2F network.

There exist a few quantitative discrepancies between results obtained from these two methods, such as the relative ranks (based on occurrence probabilities) of the minimum motifs. These discrepancies can be accounted by mathematical formalisms. Yao *et al.* use different formalisms to describe multiplicative and additive relations among links; we employ a single form (Wilson-Cowan) but use variations of parameter values to reflect different regulation modes. Also, in their ITS study Yao *et al.* allow the coexistence of two links of opposite signs from one node to another node (e.g., link 4 and 5 coexisting from EE to RP, Figure 1 of [13]). Unlike the ITS method, the Wilson-Cowan formalism used in this work does not treat multiple links between two nodes individually. Instead, we consider one effectively “lumped” link from one node to another node. We also note that the CPSS method can be implemented using other mathematical formalisms, such as the mass-action type function form. Some of these formalisms may not allow the use of a single expression to describe both activation and inhibition, and thus separate function forms are needed.

In summary, the present work and the earlier one of Yao *et al.* [13] study the key network structures underlying resettable bistability in the Rb-E2F gene network using different mathematical formalisms and search algorithms. The overall agreement on the results of these two studies suggests the effectiveness of the common reverse engineering principle they use. That is, by performing a comprehensive computer search of topology and parameter spaces, key robust network structures underlying given systems properties can be identified, largely independent of the exact mathematical formalisms. Such reverse engineering principle has been successfully used to identify key network topologies responsible for various biological properties (e.g., polarity [7], adaptation [8], and bistability [13]). The CPSS approach, by avoiding the enumeration of individual topologies for the search, further extends the application of this reverse engineering principle to biological networks containing large numbers of nodes. The CPSS software package developed in this work will aid such applications and it is freely available to researchers of interest.

## Supporting Information

Information S1 Computer package for the CPSS program.
(ZIP)

Text S1 Further explanations and results of CPSS algorithm, modeling methods and statistical analysis of motifs.
(PDF)
