# Supplementary material for: Systematic Reverse Engineering of Network Topologies: A Case Study of Resettable Bistable Cellular Responses
Source: PLoS One. 2014 Aug 29;9(8):e105833. doi: 10.1371/journal.pone.0105833 (PMC4149494; doi:10.1371/journal.pone.0105833)
Supplement: Text S1 — Further explanations and results of CPSS algorithm, modeling methods and statistical analysis of motifs. (PDF) [file pone.0105833.s002.pdf]

Supporting Information

T1. Determination of optimal number of Cluster for Case I (normal) Study

Fig. S1 presents clustering results of the 'good' sample data points for Case I (*normal*) for cluster sizes 2, 3, 4, and 5 using K-mean clustering algorithm[1, 2]. By visualizing the top two principal components, we can observe clusters are nicely attached to each other, suggesting that these clusters might be sub-clusters of a single cluster. To explore this possibility and hence to determine optimal cluster number more precisely, we computed the Mean Silhouette Coefficient (MSC) value[3, 4] for each cluster, for each cluster size. The Silhouette coefficient is a numerical value assigned to each point in each cluster that indicates how well the point was clustered. This value ranges between -1 and 1, with negative values indicating that the point was poorly assigned to its cluster, and a positive value indicating that the point was properly assigned. Fig. S2 displays the variation of Mean Silhouette Coefficient value vs. cluster number. The MSC value decreases with increase of the cluster number and it is highest for cluster number is 2. Since the minimum cluster size from the K-means clustering algorithm is 2, next we explore the possibility that the two clusters actually belongs to one big cluster. To assess this, we further check whether the parameter sets are indeed distributed in two distinct clusters or they form a single cluster by a manual inspection. First, we separate the good parameter sets for each cluster. Then, we examine the pair distribution of each combination of the 6 parameters. For most links (group 1, links 3, 7, 8, 9) the distributions are completely merges each other. An example is shown in Fig. S3a. For link 4 and 6 (group 2), clearly the K-mean clustering algorithm artificially divide a continuous distribution into two clusters (Fig. S3b). This can also been seen from the distribution between a link from group 1 and a link from group 2 (Fig. S3c). Therefore these two clusters should be merged into a single cluster. We follow a similar treatment for Case I (*constrained*) situation also (results not shown here) and found that all '*good*' parameters are distributed in a single cluster.

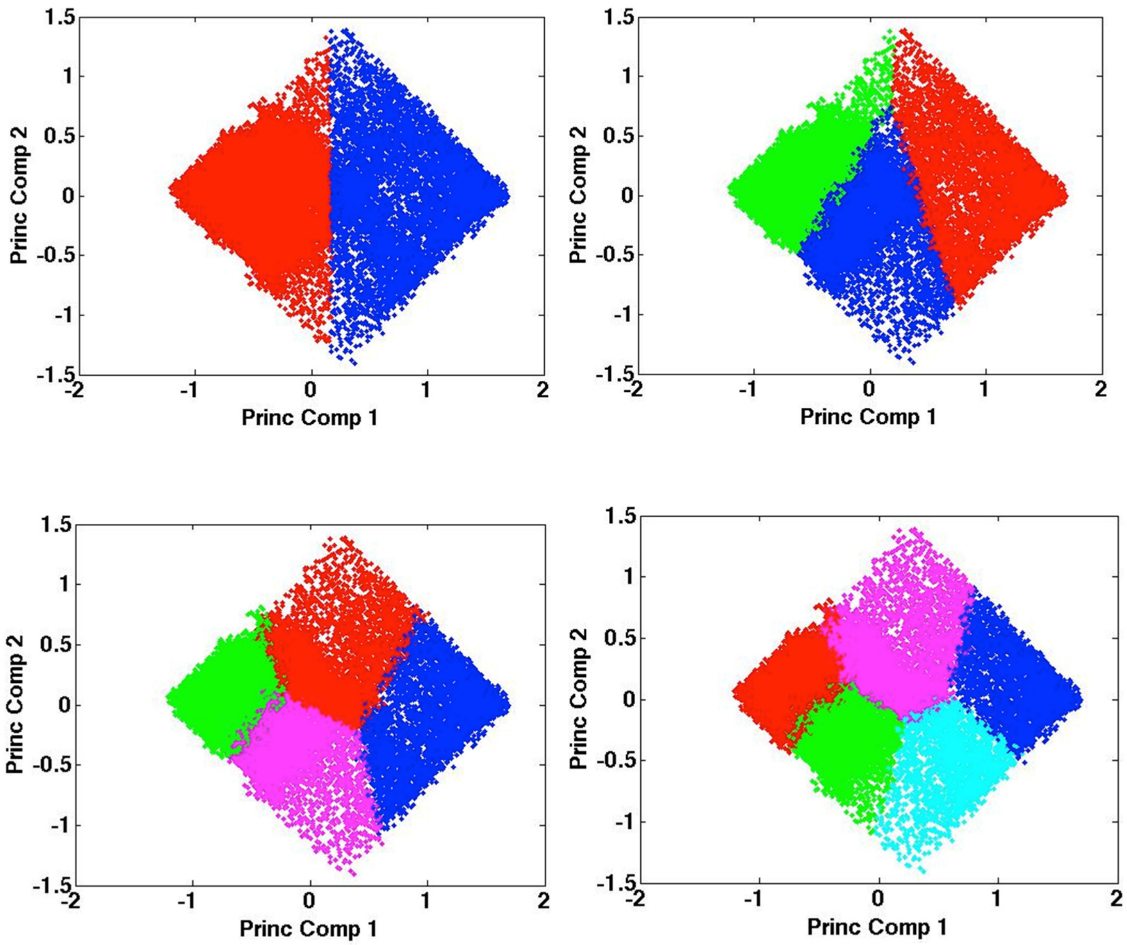

Fig. S1: Clustering results for cluster sizes 2, 3, 4, and 5. The top two principal components (PCA) of the 6 parameters are displayed (for Case I (normal) study).

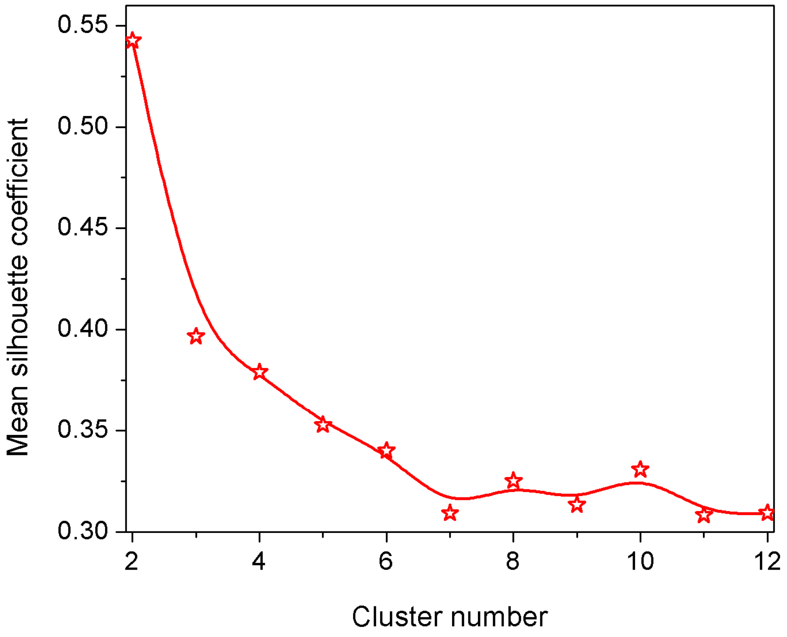

Fig. S2: Variation of Mean Silhouette coefficient value vs. cluster number for Case I (*normal*).

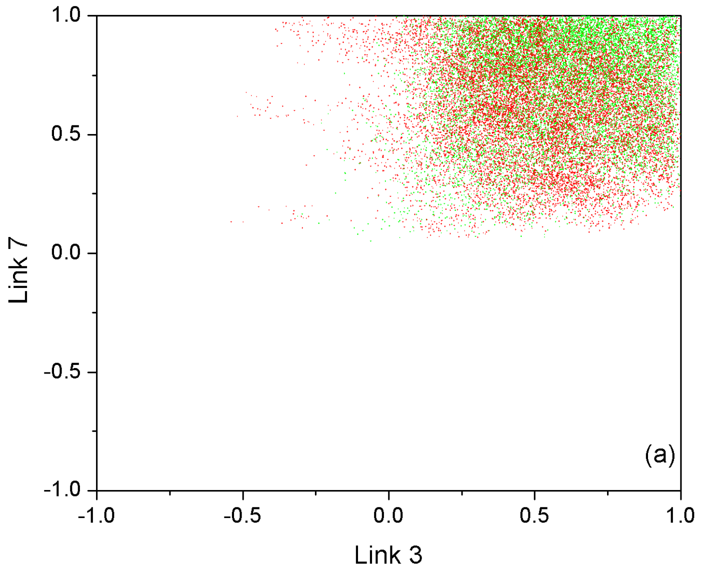

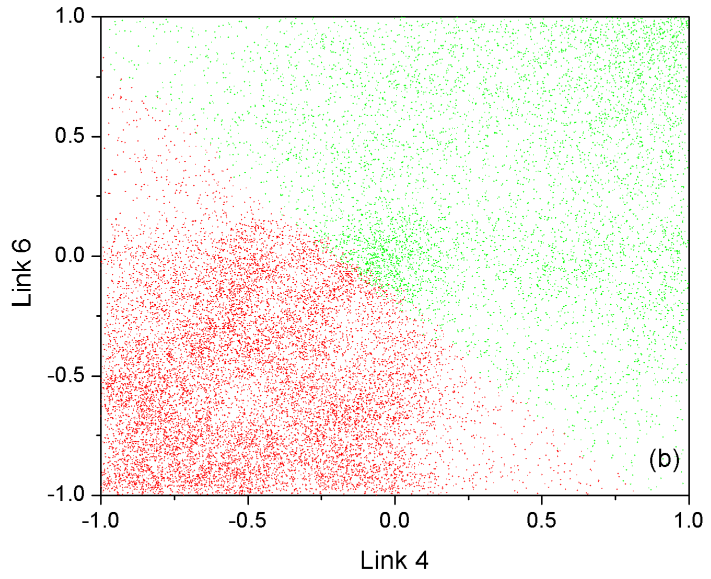

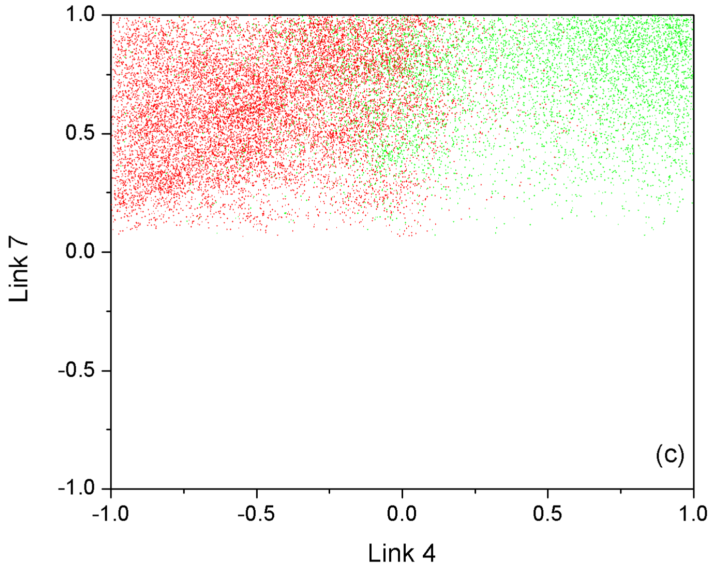

Fig. S3 pairwise distribution of good sample points between selected parameter pairs. (a) links 3-7. (b) links 4-6. (c) links 4-7.

T2. Parameter Search result for Case II (*normal*)

We perform another case study where we have considered all nine possible links (including links 1, 2 and 5 in Fig. 2 (b)) among three nodes. We use a similar two-stage

Metropolis algorithm to generate 'good' sample data points. We take 42 'good' points distributed in space from stage I search and perform 10^7 random iterations for stage II searching method, starting from each 'good' point. Finally, we get ~2.43×10^5 good sample data points distributed in the parameter space. Further analysis shows that these sample points form a single cluster.

(a) Mean Network topology for Case II (normal) situation:

The mean value matrix (Fig. S4 (a)) and the topology matrix (Fig. S4 (b)) suggest that the self-interaction strength for both MD and RB nodes, i.e.; link 1 and link 5 respectively are very weak and thus those links do not present in the mean network topology (Fig. S4(c)). This implies that these two links are not needed to obtain a resettable bistability. On the other hand our search shows that an inhibition from RB node to MD node (link 2) appears in the mean network topology. Thus link 2 and link 4 form a double negative feedback loop. This feature from the full model search was absent in the work of Yao et. al. [5], where the links 1, 2, and 5 are not considered.

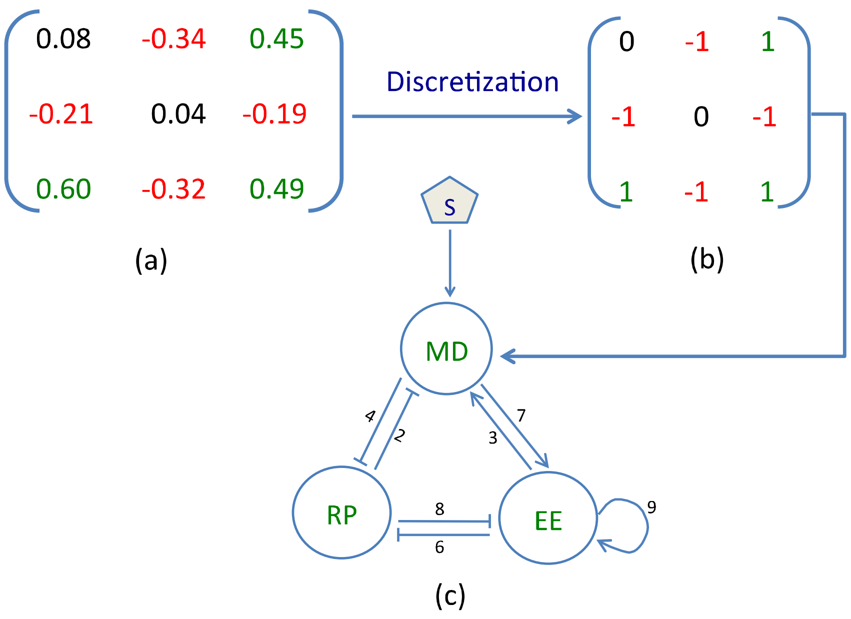

Fig. S4: Statistical method used to identify network motifs from resettable bistability data. (a) Mean value matrix consisting all nine links for Case I (normal) network search strategy. (b) Topology matrix after discretization of mean value

matrix indicating nature of the each link. (c) Mean network topology, which is responsible for Case II (normal) searching situation.

T3. Parameter Search result for Case II (Constrained) situation:

Similar to our Case I study in the main text, we have performed the parameter search with another constrain (R^0>3) on the resettable bistability. We refer this study as Case II (*constrained*) situation. The mean network topology in Fig. S5 shows that the additional constrain does not change the mean motif obtained in the '*normal*' situation Fig. S4 A comparison of MV matrix between the *normal* and *constrained* case suggest that the interaction strength of most of the links present in the mean network topology has been increased in the later situation.

(a) General Network topology for Case II (Constrained) situation

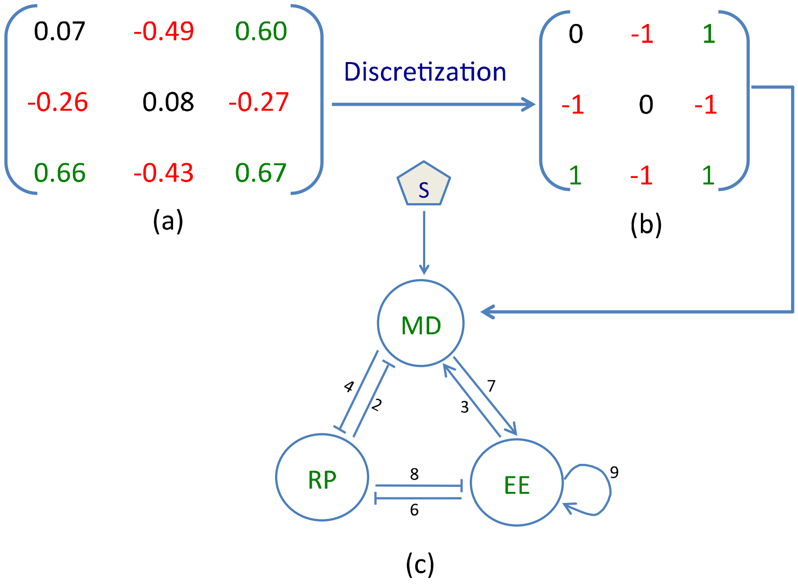

Fig. S5: Statistical method used to identify network motifs from resettable bistability data. (a) Mean value matrix consisting all eight links for Case II (constrained) network search strategy. (b) Topology Matrix after discretization of Mean value matrix indicating nature of the each link. (c) Mean network topology, which is responsible for Case II (constrained) searching situation.

T4. Backbone motif from CV matrix for Case II situation:

We construct the CV matrix for both normal (Fig. S6 (a)) and constrained (Fig. S6 (b)) situation and set a cut-off 0.8 and 0.5 respectively to determine the backbone motif. For both situation we found links 7 and 9 (Fig. S7) construct the backbone motif to generate resettable bistability. Table S1 describes the sample ration we obtain for each case to determine the backbone motif from the CV matrix. Table S2 gives a few top minimum motifs with 2, 3, or 4 links.

$$\begin{pmatrix} 6.19 & 1.17 & 0.92 \\ 2.72 & 10.19 & 3.11 \\ 0.39 & 1.27 & 0.77 \end{pmatrix}$$

(a)

$$\begin{pmatrix} 8.20 & 0.80 & 0.52 \\ 2.09 & 6.50 & 2.23 \\ 0.34 & 0.75 & 0.46 \end{pmatrix}$$

(b)

Fig. S6: Coefficient variation (CV) matrix for (a) normal situation and (b) constrained situations.

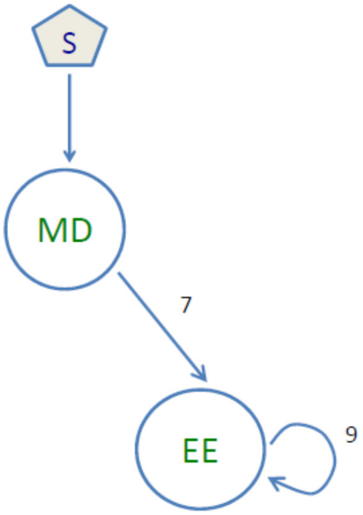

Fig. S7 Backbone motif obtained from coefficient variation (CV) matrix study for both normal situation and constrained situations.

Table S1

Case Study	Cut off Value of CV	Resultant Links	Ratio of Samples
Case II (Normal)	0.80	7, 9	0.92
Case II (Constrained)	0.50	7, 9	0.96

T5. Minimal topologies with high and moderately high occurrence probability for Case II

Table S2

(a) Minimal Model with two links

Nature of the Link	Model Situation	Occurrence Probability	Whether Present as Minimal model in [13]?
7----- 9	Case II (normal)	91.5	Yes As 7----9a or 7----9b
	Case II(Constrained)	95.8	
3 ----- 7	Case II (normal)	83.0	Yes As 2----7
	Case II (Constrained)	95.5	

(b) Minimal Model with three links

Nature of the Link	Model Situation	Occurrence probability	Whether Present as Minimal model in [13]?
2---6---7	Case II (normal)	51.7	Not Applicable
	Case II (Constrained)	62.1	
2---4---7	Case II (normal)	52.7	Not Applicable
	Case II (Constrained)	63.1	
3---4---8	Case II (normal)	54.0	Yes As 2---5---6
	Case II (Constrained)	64.7	

4---8---9	Case II (normal)	52.5	Yes
	Case II (Constrained)	66.0	As 3---6---9
4---6---8	Case II (normal)	48.1	Yes
	Case II (Constrained)	60.2	As 3---5---6
6---7---8	Case II (normal)	56.6	Yes
	Case II (Constrained)	67.9	As 5---6---7

(c) A few Minimal Model with four links:

Nature of the Link	Model Situation	Occurrence probability	Whether Present as Minimal model in [13]?
2---6---7---9	Case II (normal)	42.1	Not Applicable
	Case II (Constrained)	59.8	
2---6---8---7	Case II (normal)	43.1	Not Applicable
	Case II (Constrained)	58.7	
2---6---3---7	Case II (normal)	47.5	Not Applicable
	Case II (Constrained)	58.1	
2---4---6---7	Case II (normal)	40.3	Not Applicable
	Case II (Constrained)	54.2	
2---4---8---7	Case II (normal)	45.1	Not Applicable
	Case II (Constrained)	59.4	
4---6---8---7	Case II (normal)	48.1	Yes
	Case I (Constrained)	60.2	As 3---5---6--7
4---9---8---7	Case II (normal)	52.5	Yes
	Case II (Constrained)	66.0	As 3---6 --- 7---9
4---8---3---7	Case II (normal)	55.0	Yes
	Case II (Constrained)	64.7	As 2---3---6---7

References:

1. MacQueen, J. *Some methods for classification and analysis of multivariate observations*. in *Proceedings of the fifth Berkeley symposium on mathematical statistics and probability*. 1967. California, USA.
2. Spath, H., *The cluster dissection and analysis theory fortran programs examples*. 1985: Prentice-Hall, Inc.
3. Kaufman, L. and P.J. Rousseeuw, *Finding groups in data: an introduction to cluster analysis*. Vol. 344. 2009: Wiley. com.
4. Rousseeuw, P.J., *Silhouettes: A graphical aid to the interpretation and validation of cluster analysis.* Journal of Computational and Applied Mathematics, 1987. **20**(0): p. 53-65.
5. Yao, G., et al., *A bistable Rb–E2F switch underlies the restriction point.* Nature cell biology, 2008. **10**(4): p. 476-482.
